# Mortality Following Hip Fracture Surgery During COVID-19 Pandemic Compared to Pre-COVID-19 Period: A Case Matched Cohort Series

**DOI:** 10.5704/MOJ.2107.016

**Published:** 2021-07

**Authors:** C De, PK Harbham, C Postoyalko, B Bhavanasi, V Paringe, K Theivendran

**Affiliations:** Department of Trauma and Orthopaedics, Sandwell and West Birmingham Hospitals NHS Trust, Birmingham, United Kingdom

**Keywords:** 30-day mortality, COVID-19, pandemic, hip fracture, pre-COVID-19

## Abstract

**Introduction:**

This study aims to report on clinical outcomes and 30-day mortality of patients with neck of femur fracture during COVID-19 pandemic and compare the outcomes in a cohort during the same period prior to the pandemic.

**Material and Methods:**

The study included 66 patients with hip fracture over the age of 60 years, presented between 1st March and 15th May 2020 and matched with the patients with hip fractures (75 patients) managed during the corresponding period in 2019 as control. Data was collected on demographics, comorbidities, COVID-19 status, procedures and mortality and complications.

**Results:**

Thirty-day mortality following hip surgery was 13.6% during COVID-19 pandemic with all the mortalities in patients with ASA Grade 3 and 4. Mortality was considerably high for intracapsular fracture (20%) but highest in cemented hemiarthroplasty (20%). One third of the hip fractures operated in COVID-19 designated theatre died within 30 days of surgery. Thirty-day mortality rate for COVID-19 positive hip fracture patients were 55.5%. There has been higher 30-day mortality for hip surgeries during COVID-19 pandemic with positive correlation between patient’s COVID-19 test status and 30-day mortality following hip surgeries.

**Conclusion:**

There is strong association between 30-day mortality and the designated theatre (Clean/COVID) where the patients were operated on with higher mortality for intracapsular neck of femur fractures with significant mortality associated with cemented hemiarthroplasty particularly among symptomatic or COVID-19 positive patients. Therefore, adoption of a multidisciplinary approach is recommended to optimally balance the risk-benefit ratio for planning of management of hip fractures while considering patient’s peri-operative outcomes.

## Introduction

Severe acute respiratory syndrome coronavirus-2 (SARS-CoV-2) was declared as a pandemic on March 11, 2020 by WHO and by now it has spread to most part of the world^1^. The pandemic has tested the resilience of health-care systems, including hospitals, which were largely unprepared for the scale of the pandemic^2^. Patients having surgery are a vulnerable group at risk of SARS-CoV-2 (COVID-19) exposure in hospital and might be particularly susceptible to subsequent post-operative complications, due to the pro-inflammatory cytokine and immunosuppressive responses to surgery^3,4^. Evidence of the safety of performing surgery in COVID-19 exposed hospitals is urgently needed.

Guidelines have been published for the management of surgical patients during the COVID-19 pandemic^5,6,7^ but they are based on limited evidence. Ours is a busy district general hospital which serves more than 500,000 people^8^ in the West Midlands region, delivering its acute trauma and orthopaedic services from two sites. The West Midlands region was considered a hotspot for COVID-19 infection with second highest mortality to London^9^. Therefore, the impact of COVID-19 on perioperative complications and mortality needs to be established locally in order to enable surgical teams and patients to make evidence-based decisions during this unprecedented pandemic.

The primary aim of this study is to report on clinical outcomes and 30-day mortality rate for neck of femur fracture during COVID-19 pandemic.

## Materials and Methods

This prospective observational study included all consecutive hip fracture patients over the age of 60 years managed between 1st March and 15th May 2020 (COVID-19 cohort). We collected data from the National Hip Fracture Database (NHFD) dataset and from our electronic health records (Cerner). We also collected the demographic data from the same monthly period (1st March to 15th May) in 2019 (pre-COVID-19 cohort) and analysed any demographic differences including age, sex, Nottingham Hip Fracture Score (NHFS), American Society of Anaesthesiologists (ASA) Grade, type of hip fractures and surgical procedures between these two periods. We calculated frailty scores of the patients based on their pre-injury mobility and the level of assistance required. For all the patients, Nottingham Hip Fracture score (NHFS) was calculated and documented. This study was approved and confirmed by the trust’s Institutional Review Board that ethical clearance was not required for its conduct. The study was compliant with the STROBE guidelines for reporting observational studies.

Co-morbidities were categorised as numerical values of two, three or more in order to validate NHFS further. We specifically enquired about diabetes, hypertension, respiratory condition, cerebrovascular conditions, renal pathology, cardiological condition, gastrointestinal pathology, neurological conditions and malignancy. Dementia, hormone disorders like hypothyroidism, metabolic disorders, mental health disorders and other conditions which significantly affect the quality of life were considered among the co-morbidities. Operative variables included hip fracture type (Intracapsular or Extracapsular), primary procedure (cemented or uncemented hemiarthroplasty, dynamic hip screw fixation, intramedullary nailing, total hip replacement) and anaesthesia used.

Laboratory testing for COVID-19 infection was based on viral RNA detection by quantitative RT-PCR from swab sampling. Patients were considered to stay in high infection risk zone (Red ward) and planned to be operated in dedicated ‘COVID Theatre’ using standard personal protective equipment based on either or both clinical and radiological findings. Clinical diagnosis consistent with COVID-19 infection was made by a senior physician and based on clinical presentation indicative of COVID-19 infection, including cough, fever, and myalgia^10^. Radiological diagnosis was based on thorax CT, in keeping with locally implemented protocols. However, all patients suspected with clinical or radiological criteria, subsequently had laboratory testing for COVID-19 infection. Initially due to lack of testing capacity, patient without any suspected clinical or radiological symptoms were not tested. Asymptomatic and COVID-negative patients were managed in ‘Blue’ wards which were low infection risk zones and were operated in ‘Clean’ theatre under standard operational protocol as followed during pre-COVID period. However, staffs and surgeons used appropriate tested masks during the procedures. Importantly, all the hip fractures in this situation were discussed in a multidisciplinary meeting involving hip surgeons and orthogeriatric team to formulate an optimal treatment plan with consideration of the available logistics and expertise. Compliance to local guidelines was also ensured and at the same time, patients should get maximal benefit from the surgery by always keeping in mind their risk-benefit ratio.

Continuous variables were expressed as mean ± standard deviation (SD) and compared using Student’s t test. Categorical variables were expressed as percentages and compared using the Chi-squared Test. Independent variables were compared using Unpaired T-Test (Independent T-Test). All statistical tests of significance were two-tailed, and P values < 0.05 were considered statistically significant. Statistical analyses were performed using SPSS 16.0 statistics software [SPSS Inc. Chicago, IL, USA]. Kaplan Meier’s survival analysis was performed to compare the cohorts with the survival graphs.

## Results

Table I highlights that both groups were similar in terms age, sex, American Society of Anaesthesiology (ASA Grade), Nottingham Hip Fracture Score (NHFS), type of hip fractures and surgical procedures performed between the two cohort as there were no statistically significant differences (p-value >0.05) and therefore, both the cohorts were closely matched and comparable.

**Table I:** Distribution of patients in pre-COVID-19 and COVID-19 cohort with respect to sex, age, ASA grade, NHFS, type of fracture and type of surgery with P-value from corresponding suitable statistical test

Variables	Pre-COVID-19 Cohort	COVID-19 Cohort	P-Value
Total Patients	75	66	0.4907^a^
Male	20	25	
Female	55	41	
Mean Age	82.7	81.3	0.275^b^
Distribution of age (Independent T-Test)			
ASA Grade			
ASA-2	18	14	0.7754^a^
ASA-3	41	33	
ASA-4	16	19	
Nottingham Hip Fracture Score (NHFS)			
Mean	5	5	0.9652^b^
Distribution of NHFS (Independent T-Test)			
Type of hip fractures			
Intracapsular	28	30	0.6957^a^
Extracapsular	47	36	
Total	75	66	
Type of Hip Surgery			
DHS	21	16	0.7912^c^
IM Nail	26	22	
Cemented Hemiarthroplasty	14	20	
Uncemented Hemiarthroplasty	4	1	
THR	9	3	
Non-operative	1	4	
Total Surgery	75	66	

NB: ASA-American society of anaesthesiology; DHS-dynamic hip screw; IM-Intramedullary; THR-total hip replacement Statistical Tests used: (a) Chi-Square Test; (b) Independent T-Test; (c) ANOVA

**Fig. 1:**
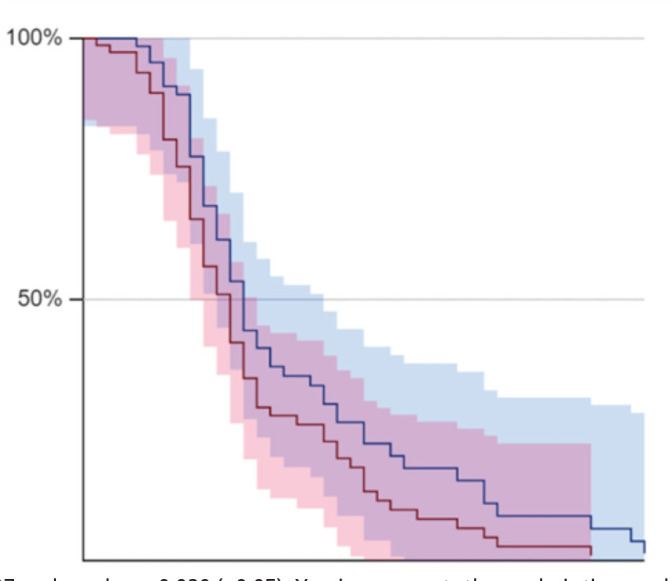
Kaplan Meier Survival graph comparing Pre-COVID-19 Cohort (red) and COVID-19 Cohort (blue)

Table II shows higher mortality in the COVID-19 cohort (13.6%) as compared to the Pre-COVID-19 cohort (5.3%) and this was statistically significant at p-value=0.039 (<0.05). Kaplan Meier Survival analysis depicts statistically significant difference in survival between the Pre-COVID-19 Cohort (red) and COVID-19 Cohort (blue) with log-rank test score z = 2.07 and p-value = 0.039(<0.05) (Fig. 1).

**Table II:** Demographic variable of the study cohort and 30 days mortality

Variables	Distribution of COVID-19 cohort			
	No of patients	30 days mortality	% 30 days mortality	P-value
Study Cohort				
Pre-COVID-19	75	4	5.3	0.039
COVID-19	66	9	13.6	
Nottingham Hip Fracture Score (NHFS)				
NHFS-1	1	0	0	
NHFS-3	6	0	0	
NHFS-4	16	0	0	
NHFS-5	18	3	16.6	0.704
NHFS-6	18	5	27.7	0.084
NHFS-7	5	1	20	0.713
NHFS-8	2	0	0	
ASA Grade				
ASA-2	14	0	0	
ASA-3	33	3	9	0.347
ASA-4	19	6	31.5	0.023
No of co-morbidities				
2	28	2	7.1	0.277
3	16	2	12.5	
>3	22	5	22.7	
Pre-injury Mobility				
Bed/Wheelchair	3	0	0	
Frame	23	4	17.4	0.573
Two stick	1	0	0	
One stick	17	4	23.5	0.241
Unaided	22	1	4.5	0.175

NB: NHFS-Nottingham hip fracture score; ASA-American society of anaesthesiology

NB: log-rank test score z = 2.07 and p-value = 0.039 (<0.05). X-axis represents the analysis time and Y-axis represents survival probability

Table II shows distribution of the patients with 30-day mortality with respect to the Nottingham Hip Fracture Score (NHFS), ASA Grading, co-morbidities and pre-injury mobility. It reflects that all the mortalities (100%) within 30 days of the surgery were amongst ASA Grade 3 and 4 with statistically significant association between ASA Grade 4 and 30-day mortality with p-value <0.05 (p value=0.023). There has been highest mortality (22.7%) for those patients having more than three co-morbidities. However, the association of comorbidities was not statistically significant (p value=0.277). In our study, we found weak association between pre-injury mobility and 30-day mortality for hip fractures among different categories with p-values >0.05 (Table II).

In our study group (Table III), we had 30 (45.5%) patients with intracapsular neck of femur fracture (IC) compared to 36 (54.5%) patients with extracapsular fracture. Although, mortality was higher for intracapsular fracture, it was not statistically significant (p value=0.169).

**Table III:** Perioperative variables of the study cohort and 30 days mortality

Variables	Distribution of COVID-19 cohort			
	No of patients	30 days mortality	% 30 days mortality	P-value
Type of Fracture				
Intracapsular (IC)	30	6	20	0.169
Extracapsular	36	3	8.3	
Type of surgery				
Cemented Hemi	20	4	20	0.321
Uncemented Hemi	1	0	0	
DHS	16	1	6.25	0.322
IM Nail	22	2	9	0.446
THR	3	0	0	
Non-operated	4	2(both IC)	50	0.028
Anaesthesia				
Regional Anaesthesia	39	3	7.7	0.496
General Anaesthesia	18	3	16.7	
Combined	5	1	20	
Theatre				
COVID	9	3	33	0.023
Clean	53	4	7.54	
Not Operated	4	2	50	0.028
Ward				
Red	9	3	33	0.043
Blue	57	6	10.5	
COVID Status				
Positive	9	5	55.5	0.008
Negative	20	2	10	
Not Tested	37	2	5.4	

NB: IC-Intracapsular; Hemi-Hemiarthroplasty; DHS-Dynamic hip screw; IM-Intramedullary; THR-total hip replacement

Out of these 6 patients with intracapsular fracture, who died within 30 days of surgery, 4 (66.6%) patients were operated with cemented hemiarthroplasty and 2 (33.3%) were not operated being medically unwell for surgery. Out of these 4 patients operated with cemented hemiarthroplasty, 3 (75%) patients were operated in ‘COVID theatre’ and treated in the ‘Red Ward’. However, this higher incidence of death following cemented hemiarthroplasty was not statistically significant (P value= 0.321). Mortalities following extracapsular fracture fixation with dynamic hip screw (P value= 0.322) or intramedullary nail (P value= 0.446) were not statistically significant indicating multifactorial causation of post-operative deaths. Although there was higher mortality following use of general anaesthesia, overall mortality was not significantly impacted (P value= 0.496) by the anaesthetic choice. Importantly, there was statistically significant mortality for conservatively managed hip fractures (P value= 0.028).

Table III shows higher mortality for patients operated in ‘COVID Theatre’ and this association was statistically significant with P value= 0.023 (<0.05). Similarly, there was higher mortality for patients who were managed in the ‘Red’ wards with statistically significant association (P value= 0.043).

Table III shows that there was 55.5% (5 out of 9) mortality for COVID-19 positive patients within 30 days of hip surgery as compared to 10% (2 out of 10) mortality for COVID-19 negative patients. Statistical analysis shows significant association between 30 days mortality and patients’ COVID-19 status (positive/negative by COVID-19 swab) with p value <0.05 (P value= 0.008).

Table IV shows further analysis of the COVID-19 with respect to peri-operative variables. The patients who died within 30 days were operated sooner (33.62hrs vs 43.06hrs) than who survived. However, the anaesthetic induction time, procedure duration and the length of hospital stay were longer for those who died within 30 days of their surgery. These could be an associated finding as these factors were not statistically significant for causation of higher mortality (P-value >0.05).

**Table IV:** Perioperative variables of the study cohort and 30-day mortality

Variable	Survivor	COVID-19 Cohort 30-Day Mortality	P-value
Mean time to surgery (Hrs)	43.06 (SD 34.16)	33.62 (SD 36.72)	0.218
Anaesthetic induction time (Min)	15.3 (SD 9.2)	24.1 (SD 19.5)	0.083
Procedure duration (Min)	77.2 (SD 17.3)	89.3 (SD 23.5)	0.067
Mean length of stay (Days)	21.7 (SD 12.75)	23.44 (SD 13.5)	0.457

NB: Independent T-Test used to calculate the P-values

When we analyse the cause of death, we found out that 6 (66.7%) out of 9 mortalities were due to pulmonary complications. Two patients (22.2%) died because of non-pulmonary complications and for one patient (11.1%) the cause of death was still under investigation. For all the patients who were COVID-19 positive and died within 30 days of their hip surgery, cause of death was pulmonary complication (100%). A total of 50% of the mortalities within 30 days of hip surgery in COVID-19 negative group was because of pulmonary complications. Considering the number of deaths within 30 days following cemented hemiarthroplasty, pulmonary complication accounted for 75% (3 out of 4) of the mortality.

## Discussion

Since 2007 the NHFD has reported a progressive improvement in mortality in the month after hip fracture. This gives an overall mortality rate of 6.7% for 2016. This is better than 7.1% 30-day mortality in 2015 and continues the steady improvement documented since 2007 when it was 10.9%. Moreover, this trend continues with just 4,007 people (6.1%) dying in 2018. This figure represents a decrease of one in eight when compared with the mortality figure of 6.9% the NHFD reported for 2017 and implies that 564 fewer people died within a month of breaking their hip in 2018. Over the seven years (2011-2017) the NHFD recorded that 7.5% of people died within 30 days. This figure varied significantly across the months of the year, ranging from just 6.7% in July to a peak of 8.7% in January^11^. In the study group, 13.6% patients died within 30 days of their hip surgery which is higher than the national statistics as published in NHFD annual report. However, during COVID-19 pandemic there might be additional impact on the overall mortality which might be reflected in the forthcoming NHFD annual report.

In our study group, 30-day mortality according to Nottingham Hip Fracture (NHFS) Score was high compared to predicted 30-day mortality scale according to the NHFS. For NHFS 5,6 and 7, we observed 16.6%,27.7% and 20% mortality, respectively within 30 days of hip surgery as compared to 6.9%, 11% and 16% predicted mortality, respectively^12^.

In the study group, patients who died within 30 days of the surgery, 33% were ASA Grade-3 and 66% were ASA Grade-4. Therefore, all the mortalities (100%) were among ASA Grade 3 and 4. There has been statistically significant association between patients higher ASA grade and 30-day mortality. However, other studies have found 85% 30-day mortality for hip fractures for patients with ASA grade 3 and 413. Our study population has experienced higher mortality in this group during COVID-19 pandemic compared to the year before the pandemic.

In the COVID-19 cohort, there has been positive correlation between comorbidities and mortality for hip surgeries for neck of femur fracture. We observed higher 30-day mortality for patients with more comorbid conditions and for patients with more than three co-morbidities, the mortality was 22.7%. Although conducted during pre-COVID-19 pandemic, other clinical studies support that comorbidities were positively associated with surgical procedure and perioperative management of elderly patients aged more than sixty years with hip fracture^14^. However, in our study we found weak association between pre-injury mobility and 30-day mortality for hip fractures. This contradicts with studies which shows about 14% and 18% mortality within 30 days of hip surgery among patients who were non-ambulatory and frame users, respectively during their pre-injury status and higher correlation with mortality and poor pre-injury mobility^15^. Therefore, it might be other factors during the COVID-19 pandemic which might have impacted the outcome.

According to NHFD-2019 annual report^11^, there was little variation in outcome for intracapsular fracture, but it found significant variation for trochanteric fractures: East of England had the highest percentage of deaths within 30 days (8.5%) and the North East the lowest (5.9%). However, in our study we experienced higher mortality for intracapsular neck of femur fractures (20%) compared to intertrochanteric fractures (8.12%). Two third of the patients with intracapsular neck of femur fractures who were operated with cemented hemiarthroplasty died within 30-day of surgery and 75% of these deaths happened when the surgery was done in dedicated ‘COVID-Theatre’. Overall, 20% of the patients who were operated with cemented hemiarthroplasty died within 30 days of their surgery. Therefore, we experienced the highest mortality for the patients with intracapsular neck of femur fracture operated with cemented hemiarthroplasty in designated ‘COVID Theatre’. For management of intertrochanteric fractures, there has been 6.25% and 9% mortality within 30 days when they were operated with DHS and intramedullary nailing, respectively. In our study cohort, pulmonary complications have accounted for 75% of the mortality within 30 days of hip surgery for COVID-19 positive patients who underwent cemented hemiarthroplasty. The exact reason for this remains unknown. We could have hypothesised that the higher mortality following cemented hemiarthroplasty in COVID-19 positive patients may be because of their higher susceptibility to bone cement implantation syndrome (BCIS) due to compromised cardiorespiratory function due to COVID-19 infection. This could act as a ‘second hit’ to an already compromised physiology. However, multifactorial issue and type of implant or cementation is just one of the multiple factors. Mortality from Bone cement implantation syndrome (BCIS) may vary widely according to the severity. Early mortality in BCIS Grade 1 (9.3%) did not differ significantly from BCIS Grade 0 (5.2%), while early mortality in BCIS Grade 2 (35%) and grade 3 (88%) were significantly higher when compared with Grades 0 and 1. ASA grade III-IV, chronic obstructive pulmonary disease and pulmonary compromise may act as an independent predictor severe BCIS and Severe BCIS was associated with 16-fold increase in mortality^16^. In our study, management of every hip fracture were discussed in multidisciplinary meeting involving hip surgeons and decisions were taken considering local guidelines, logistics and expertise available. This study was mostly oriented on overall management of hip fractures during the COVID-19 pandemic to figure out any key factors in the practice which could have contributed to the outcome.

There has been statistically significant correlation between 30-day mortality for hip surgery and in which theatre, either clean or COVID which the patients were operated in. As per our local guidelines, we continued to treat patients in ‘Red’ wards with high infection risk zone who were operated in ‘COVID’ theatre and therefore the similar mortality pattern followed for their treatment pathway. Hence, we experienced higher mortality for hip fracture surgery in the ‘Red ward’ (33%) as compared to the ‘Blue ward’(10.5%). Current evidence from the recent international COVIDSurg Collaborative group^17^ study highlighted overall 30-day mortality in their study was 23.8%, and was high across all patient subgroups with 18.9% in elective patients, 25.6% in emergency patients, 16.3% in patients who had minor surgery, and 26.9% in patients who had major surgery.

Patients’ COVID status (positive/negative by COVID swab) was significantly associated with 30-day mortality following hip surgery with statistically significant positive correlation. Among COVID positive patients with hip fracture, 55.5% died with 30 days of their hip surgery. The only study so far is a series of cases in Wuhan at an early stage of the pandemic which showed 20.5% early mortality for across speciality elective surgeries and 35% mortality for more major surgeries including hip replacement or revision procedures among patient with COVID-19 infection18. However, our study for hip fractures reflects a higher 30-day mortality compared to the current evidence.

In the study we experienced 13.6% mortality within 30 days of the hip surgery and pulmonary complications have been predominant (66.7%) cause for the mortality. Although other studies have indicated 30-day mortality following hip surgery could be 8.7%, the most common causes of death included pulmonary complications, most commonly pneumonia, and concomitant chest infection during admission increases the mortality following the hip surgery^19^. Recently published evidence from multicentre COVIDSurg collaborative study group shows that 38% mortality within 30 day of surgery and pulmonary complications accounted for 82.6% of all deaths^17^. However, in the COVIDSurg study there were no comparative (control) group and the number of hip fractures was only 115 over 24 different countries worldwide with various perioperative protocols, testing regimes and health systems. In our study, although with a smaller group, there is a direct comparison with specific perioperative protocols within our hospital avoiding bias and confounding factors.

In our study, we have noted 50% mortality for patients who were medically unwell and not fit for surgery. In a study by Moulton *et al* that mortality at 30 days and one year was 31.3% and 56.3%, respectively for patients who were managed conservatively for intracapsular neck of femur fracture^20^. The reason for the higher mortality in our series is the morbidity associated with COVID-19 infection.

A recent UK hip fracture study of 18 COVID-19 positive patients over 3 centres showed 30-day mortality rate of 22.2%^21^. However, in their multi-centres series there was no pre-COVID-19 comparative group. Our series is the only report to date that reports UK hip fractures from a single centre using a control pre-COVID-19 direct comparator group. Therefore, we are confident that the mortality in our series is causally related to COVID-19 mortality rather than other possible confounders of comorbidities, ASA grade and NHFS.

We acknowledge the limitations of a single centre study with relatively small sample size. However, this study reflected early impact of the pandemic on the management of fragility fracture. One has to be cautious about drawing strong conclusions based on this number of patients, but clear trends and observations can be noted, and this may provide helpful guidance when discussing issues of complications including mortality with patients and their family as a part of the consenting process for surgery. The data on delayed complications and revision rates were not available in the short time frame analysed.

## Conclusion

We observed a higher 30-day mortality for hip fracture patients during the COVID-19 pandemic. There has been positive association between patient’s COVID-19 test results and 30-day mortality following hip fracture surgery. Furthermore, there is strong association with 30-day mortality and in which theatre, Clean or COVID the patients were being operated on. This study also observed higher mortality for intracapsular neck of femur fractures with significant mortality associated with cemented hemiarthroplasty particularly among symptomatic or COVID-19 positive patients. Therefore, adoption of multidisciplinary approach is recommended to optimally balance the risk-benefit ratio for planning of management of hip fractures considering perioperative outcomes.

## References

[1] World Health Organization (WHO): WHO announces COVID-19 outbreak a pandemic. (2020). http://www.euro.who.int/en/health-topics/healthemergencies/coronavirus-covid-19/news/news/2020/3/whoannounces-covid-19-outbreak-a-pandemic.

[2] Horton R (2020). Offline: COVID-19 and the NHS-“a national scandal”.. Lancet..

[3] Besnier E, Tuech JJ, Schwarz L (2020). We asked the experts: Covid-19 outbreak: is there still a place for scheduled surgery? “Reflection from pathophysiological data”.. World J Surg..

[4] Huang C, Wang Y, Li X, Ren L, Zhao J, Hu Y (2020). Clinical features of patients infected with 2019 novel coronavirus in Wuhan, China.. Lancet..

[5] Coccolini F, Perrone G, Chiarugi M, Marzo FD, Ansaloni L, Scandroglio I (2020). Surgery in COVID-19 patients: operational directives.. World J Emerg Surg..

[6] COVIDSurg Collaborative. (2020). Global guidance for surgical care during the COVID-19 pandemic.. Br J Surg..

[7] Tao KX, Zhang BX, Zhang P, Zhu P, Wang GB, Chen XP (2020). Recommendations for general surgery clinical practice in novel coronavirus pneumonia situation.. Zhonghua Wai Ke Za Zhi..

[8] Sandwell and West Birmingham Hospitals. (2019). http://https://www.swbh.nhs.uk/wp-content/uploads/2019/03/ED-Annual-Equality-Report-January-2019-FINAL.pdf.

[9] Office for National Statistics. Coronavirus (COVID-19). Latest data and analysis on coronavirus (COVID-19) in the UK and its effect on the economy and society.. http://https://www.ons.gov.uk/peoplepopulationandcommunity/healthandsocialcare/conditionsanddiseases.

[10] Zhou F, Yu T, Du R, Fan G, Liu Y, Liu Z (2020). Clinical course and risk factors for mortality of adult inpatients with COVID-19 in Wuhan, China: a retrospective cohort study.. Lancet..

[11] Royal College of Physicians. (2019). http://https://www.nhfd.co.uk/files/2019ReportFiles/NHFD_2019_Annual_Report.pdf.

[12] Moppett IK, Parker M, Griffiths R, Bowers T, White SM, Moran CG (2012). Nottingham Hip Fracture Score: longitudinal and multi-centre assessment.. Br J Anaesth..

[13] Yeoh CJC, Fazal MA. (2014). ASA Grade and Elderly Patients with Femoral Neck Fracture.. Geriatr Orthop Surg Rehabil..

[14] Wei J, Zeng L, Li S, Luo F, Xiang Z, Ding Q (2019). Relationship between comorbidities and treatment decision-making in elderly hip fracture patients.. Aging Clin Exp Res..

[15] Faraj AA, Patil V. (2006). Correlation Between Pre-Injury Mobility and ASA Score With the Mortality Following Femoral Neck Fracture in Elderly.. Eur J Orthop Surg Traumatol..

[16] Olsen F, Kotyra M, Houltz E, Ricksten SE (2014). Bone Cement Implantation Syndrome in Cemented Hemiarthroplasty for Femoral Neck Fracture: Incidence, Risk Factors, and Effect on Outcome.. Br J Anaesth..

[17] COVIDSurg Collaborative. (2020). Mortality and pulmonary complications in patients undergoing surgery with perioperative SARS-CoV-2 infection: an international cohort study.. Lancet..

[18] Lei S, Jiang F, Su W, Chen C, Chen J, Mei W (2020). Clinical characteristics and outcomes of patients undergoing surgeries during the incubation period of COVID-19 infection.. EClinicalMedicine..

[19] Sheikh HQ, Hossain FS, Aqil A, Akinbamijo B, Mushtaq V, Kapoor H (2017). A Comprehensive Analysis of the Causes and Predictors of 30-Day Mortality Following Hip Fracture Surgery.. Clin Orthop Surg..

[20] Moulton LS, Green NL, Sudahar T, Makwana NK, Whittaker JP (2015). Outcome after conservatively managed intracapsular fractures of the femoral neck.. Ann R Coll Surg Engl..

[21] Archer JE, Kapor S, Piper D, Odeh A (2020). The impact of COVID-19 on 30-day mortality in patients with neck of femur fractures.. Bone Jt Open.

